# Supplementary figures and images for: Histone demethylase AMX-1 is necessary for proper sensitivity to interstrand crosslink DNA damage
Source: PLoS Genet. 2021 Jul 30;17(7):e1009715. doi: 10.1371/journal.pgen.1009715 (PMC8357103; doi:10.1371/journal.pgen.1009715)

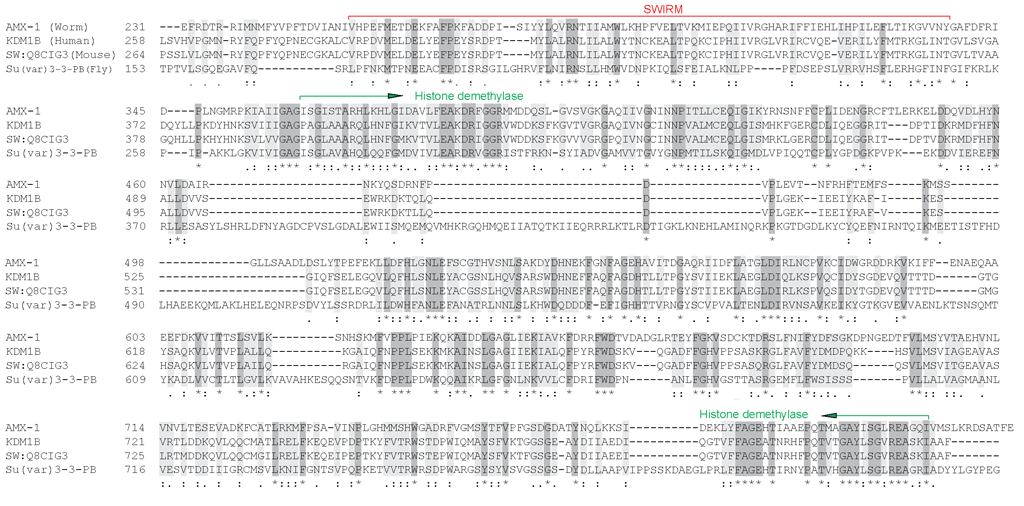

Supplement: S1 Fig — AMX-1 (C. elegans CELE_B0019.1), KDM1B (humans), SW:Q8CIG3 (mouse) and Su(var)3-3-PB (fly) were aligned using the Uniplot Clustal O multiple sequence alignment tool. SWIRM and histone demethylase domains are indicated. (*) denotes a single, fully conserved residue; (:) indicates conservation between groups of highly similar properties and (.) represents conservation between groups of weakly similar properties. (TIF) [file pgen.1009715.s001.tif]

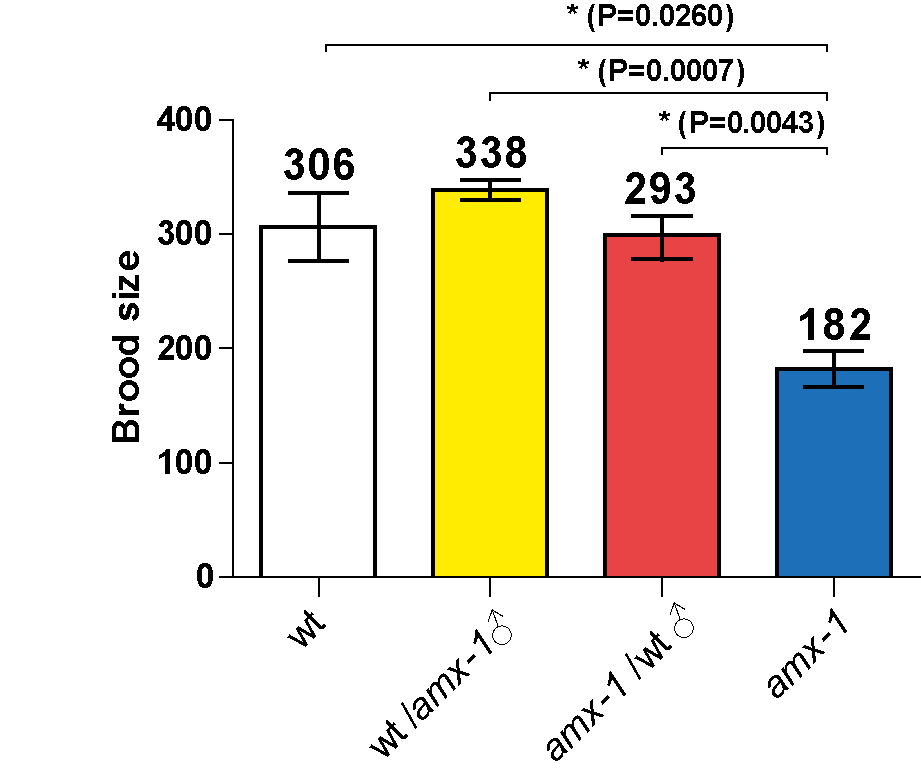

Supplement: S2 Fig — Brood size is scored among the progeny of worms of the indicated genotypes. Error bars represent the standard error of the mean. N = 24 for each genotype. An asterisk indicates a statistically significant reduction compared to wild type by the two-tailed Mann-Whitney test, 95% C.I. (TIF) [file pgen.1009715.s002.tif]

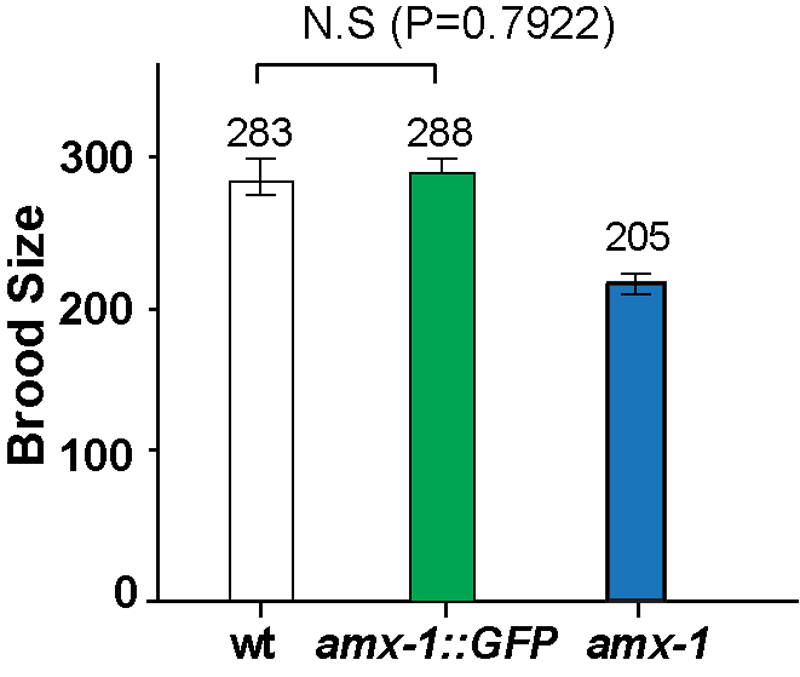

Supplement: S3 Fig — The brood size of amx-1 mutants is significantly decreased compared to wild type (P = 0.0087) while it is not altered for amx-1::GFP worms (P = 0.7922). (TIF) [file pgen.1009715.s003.tif]

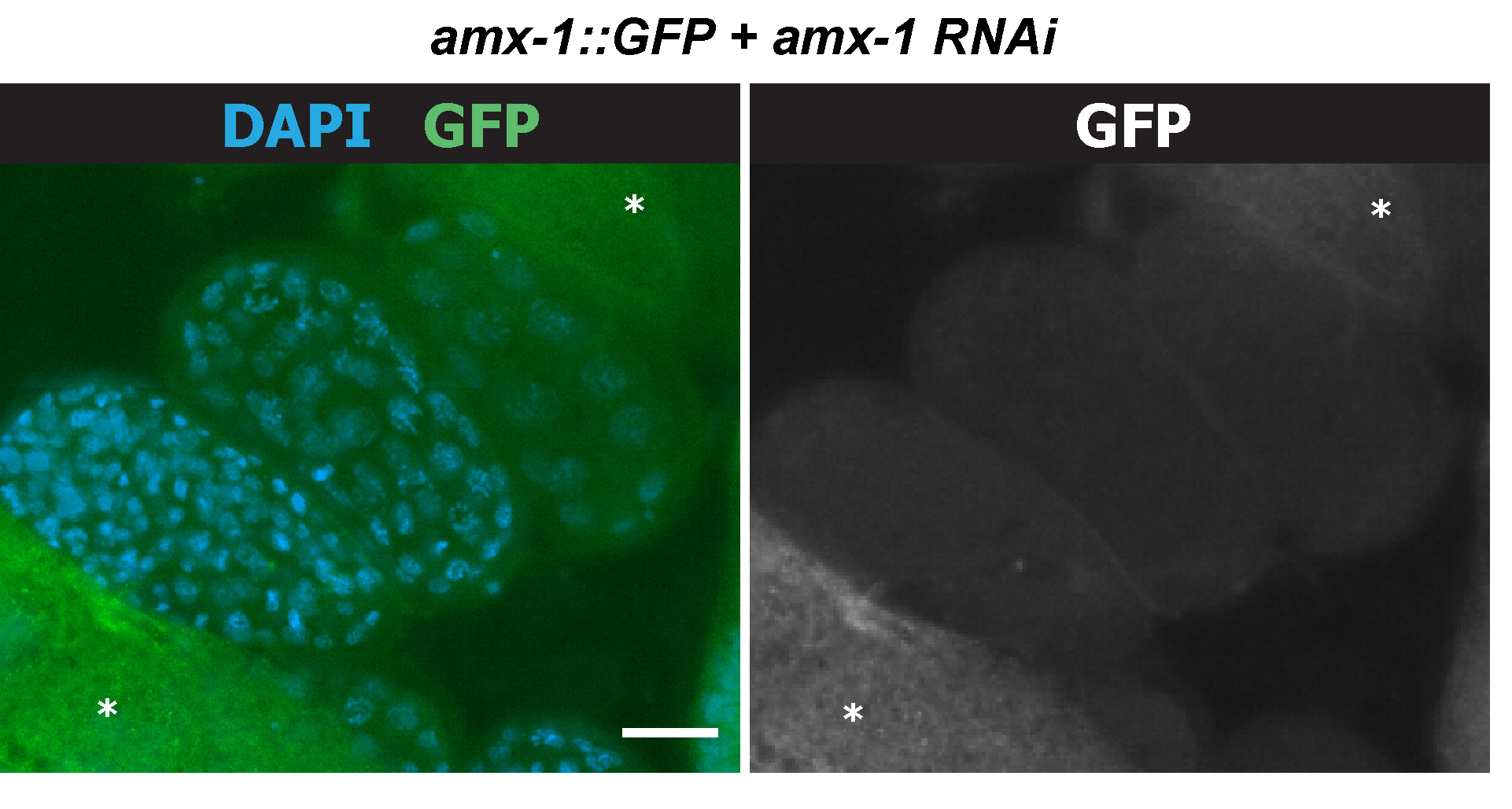

Supplement: S4 Fig — Asterisks indicate gut autofluorescence. Bar = 10μm. (TIF) [file pgen.1009715.s004.tif]

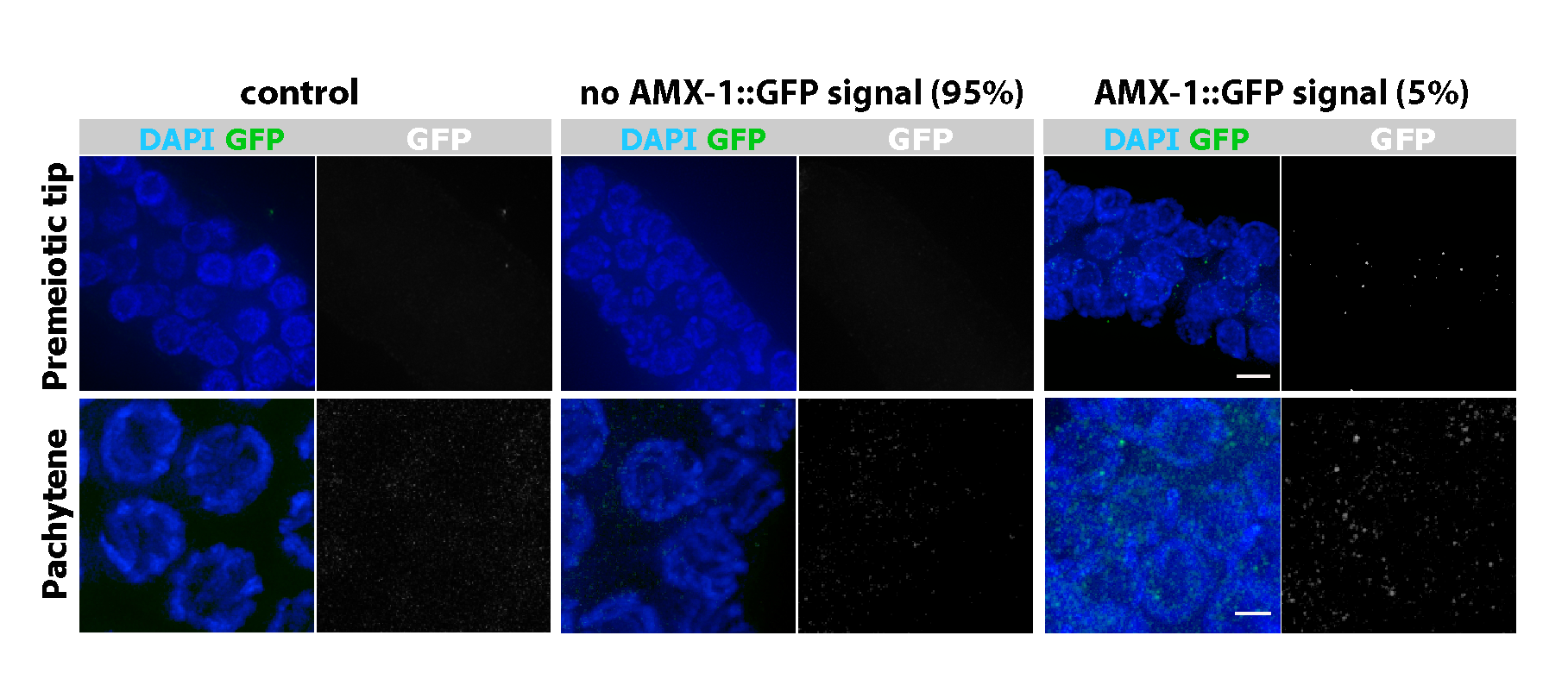

Supplement: S5 Fig — We observed 5% of gonads containing AMX-1::GFP signal either in the premeiotic tip or pachytene stage (5 out of 100 and 5 out of 96, respectively). Wild type (N2) is used as a control. P = 0.032 in control versus AMX-1::GFP in the premeiotic tip, and P = 0.0248 in control versus AMX-1::GFP in pachytene, by the two-tailed t-test. Bars = 10μm. (TIF) [file pgen.1009715.s005.tif]

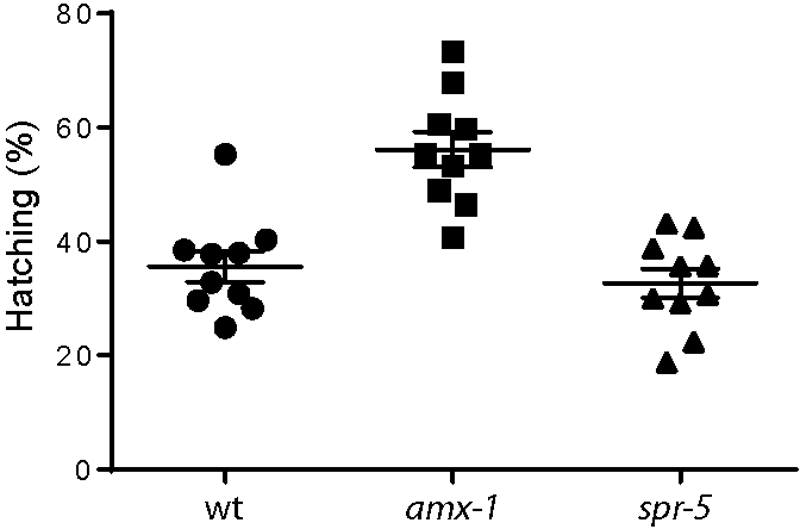

Supplement: S6 Fig — N = 70–80 worms. (TIF) [file pgen.1009715.s006.tif]

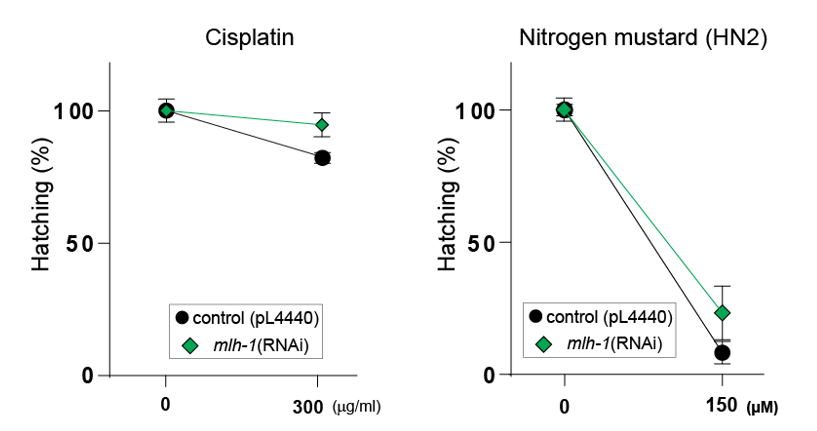

Supplement: S7 Fig — 8% vs 24% at 150 μM HN2, P = 0.0003, and 82% vs 95% at 300 μg/ml cisplatin, P<0.0001, by the two-tailed Mann-Whitney test, 95% C.I. (TIF) [file pgen.1009715.s007.tif]

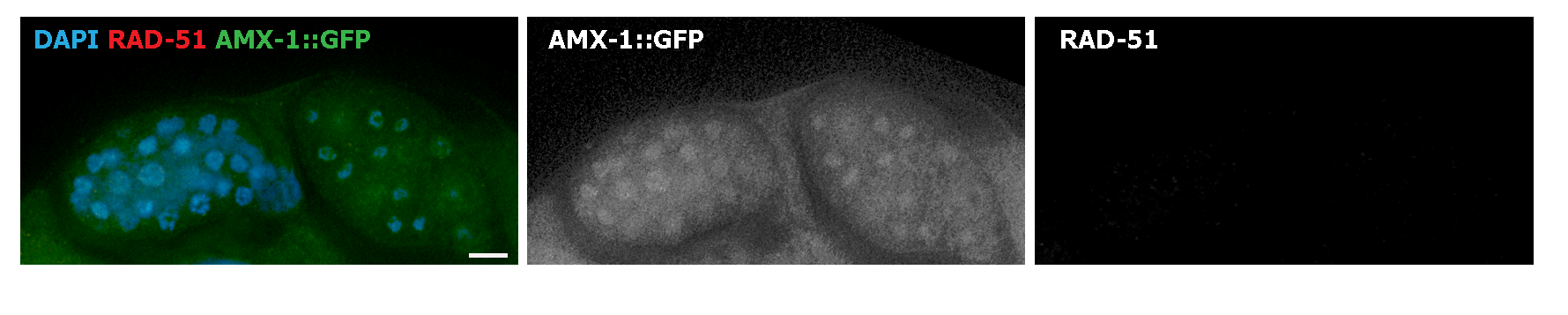

Supplement: S8 Fig — No distinct RAD-51 foci were observed in embryos in wild type control. amx-1::GFP animals were treated with 150 μM of HN2 in NGM containing E. coli OP50 for 20 hours. Higher magnification image is shown as inset. Bar = 2 μm. (TIF) [file pgen.1009715.s008.tif]

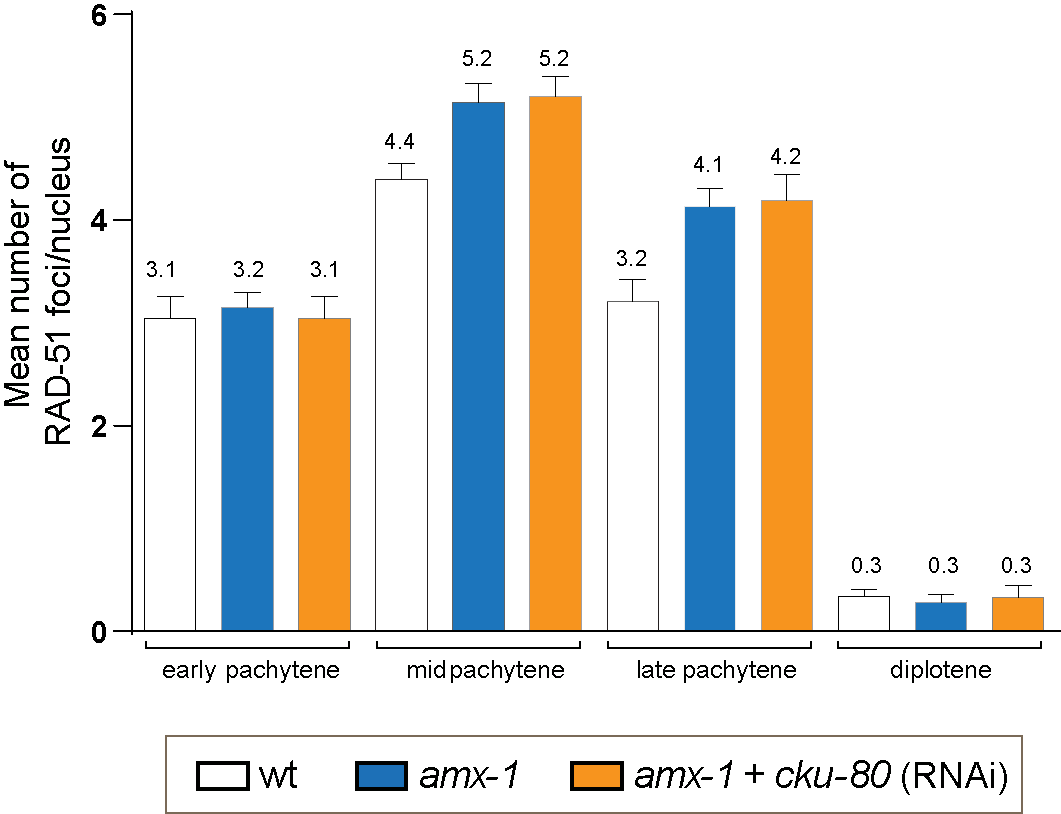

Supplement: S9 Fig — Mean numbers of RAD-51 foci observed per nucleus on each zone along the germline axis (x-axis). P = 0.8888 in amx-1 and amx-1 + cku-80 (RNAi) in early pachytene. P = 0.1763 in amx-1 and amx-1 + cku-80 (RNAi) in mid pachytene. P = 0.5509 in amx-1 and amx-1 + cku-80 (RNAi) in late pachytene. P = 0.2431 in amx-1 and amx-1 + cku-80 (RNAi) in diplotene by the two-tailed Mann–Whitney test, 95% C.I. Error bars represent standard error of the mean. (TIF) [file pgen.1009715.s009.tif]

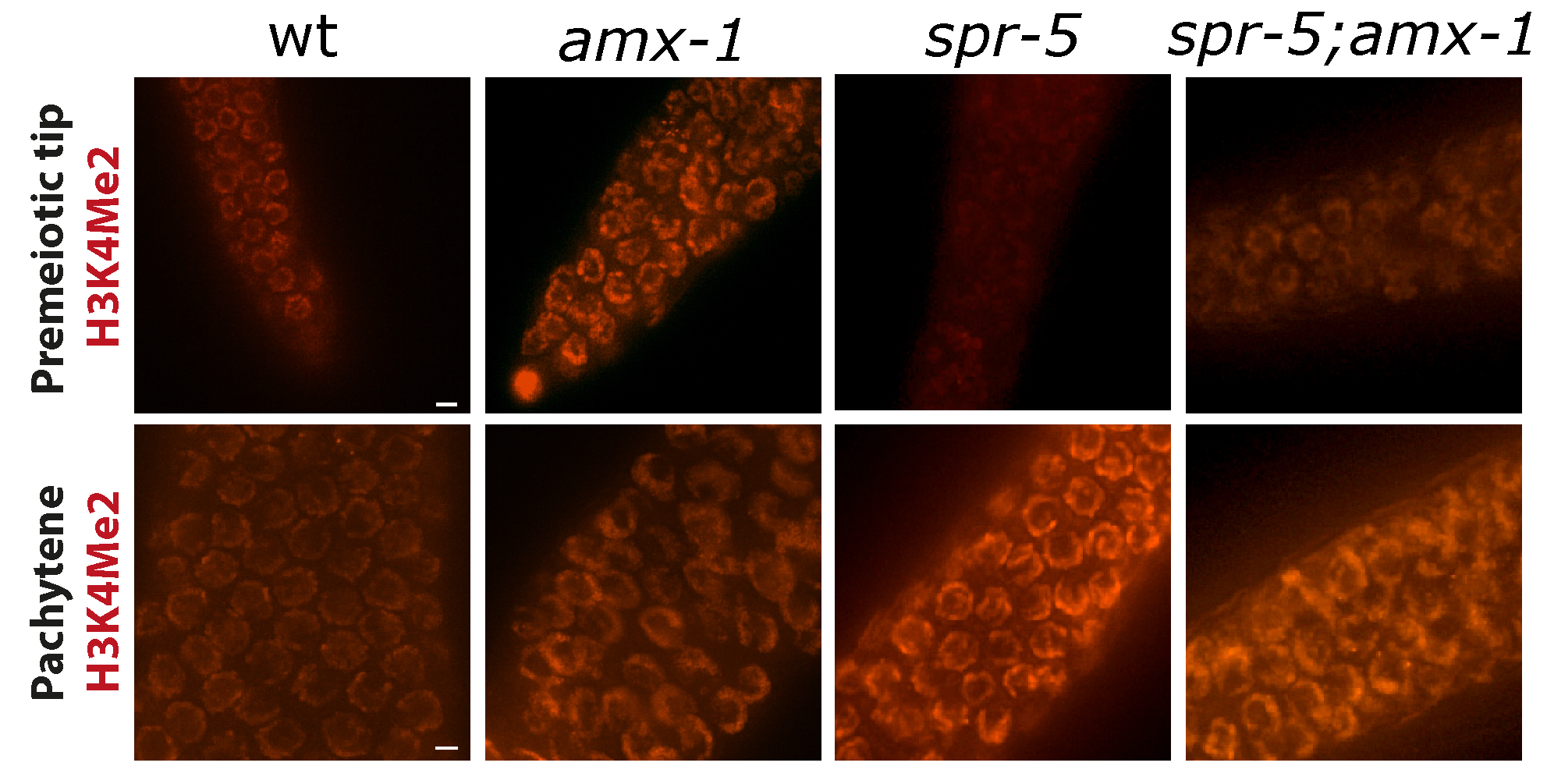

Supplement: S10 Fig — Premeiotic tip and pachytene stage nuclei are shown for the indicated genotypes. Bars, 2 μm. (TIF) [file pgen.1009715.s010.tif]
