# Supplementary material for: Histone demethylase AMX-1 is necessary for proper sensitivity to interstrand crosslink DNA damage
Source: PLoS Genet. 2021 Jul 30;17(7):e1009715. doi: 10.1371/journal.pgen.1009715 (PMC8357103; doi:10.1371/journal.pgen.1009715)

**S1 Table.** Number of gonads exhibiting AMX-1::GFP signal observed in this study for the indicated genotypes.


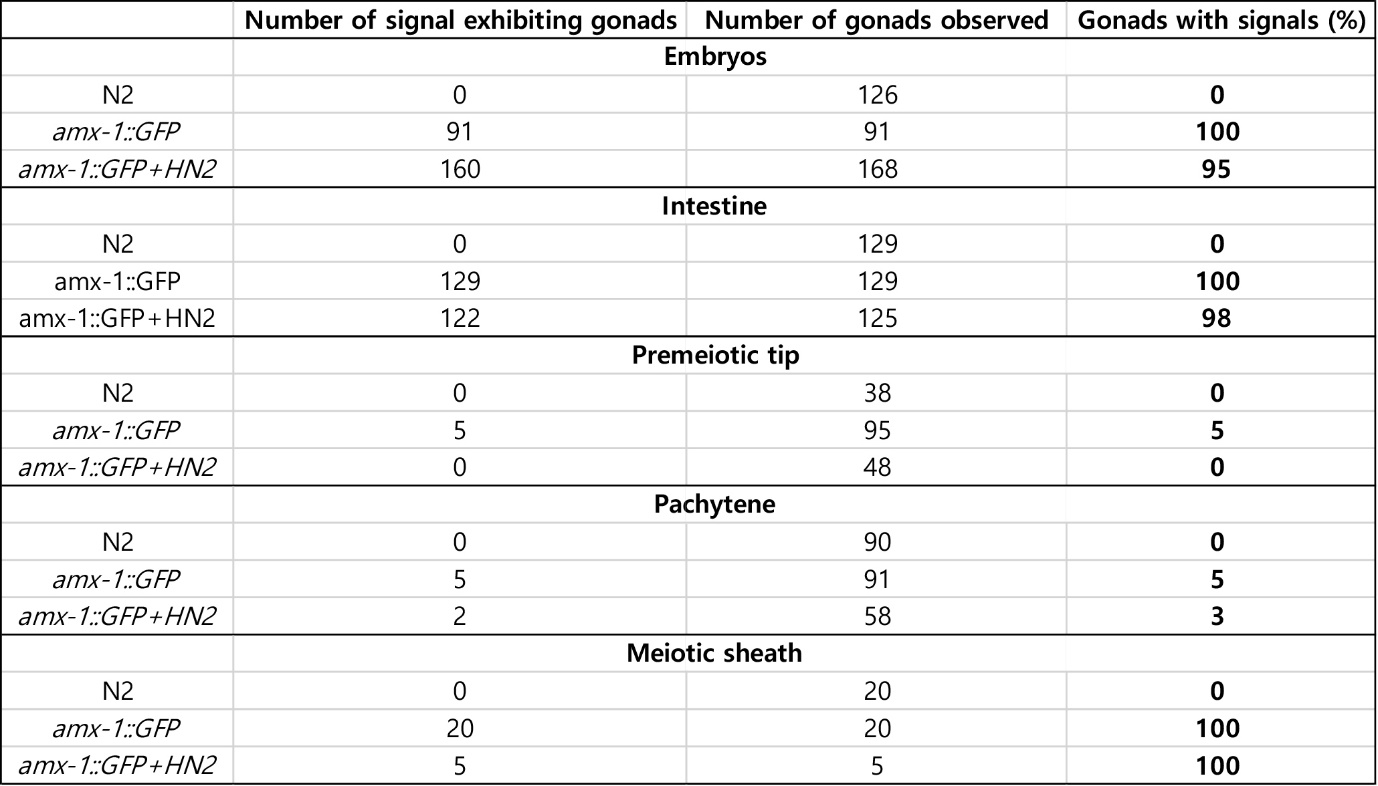

Supplement: S1 Table — (DOCX) [file pgen.1009715.s011.docx]
