# Supplementary material for: Histone demethylase AMX-1 is necessary for proper sensitivity to interstrand crosslink DNA damage
Source: PLoS Genet. 2021 Jul 30;17(7):e1009715. doi: 10.1371/journal.pgen.1009715 (PMC8357103; doi:10.1371/journal.pgen.1009715)
Supplement: S2 Table — (DOCX) [file pgen.1009715.s012.docx]

**S2 Table.** Number of gonads containing pCHK1 signal observed in this study for the indicated genotypes.

|  | **Embryos** | **Premeiotic tip** | | **Pachytene** |
| --- | --- | --- | --- | --- |
| N2 | 2 out of 25 (8 %) | | 0 out of 12 gonads (0 %) | 0 out of 12 gonads (0 %) |
| *amx-1* | 10 out of 36 (28 %) | | 9 out of 18 gonads (50 %) | 12 out of 18 gonads (66 %) |
