# Supplementary material for: Histone demethylase AMX-1 is necessary for proper sensitivity to interstrand crosslink DNA damage
Source: PLoS Genet. 2021 Jul 30;17(7):e1009715. doi: 10.1371/journal.pgen.1009715 (PMC8357103; doi:10.1371/journal.pgen.1009715)
Supplement: S3 Table — (DOCX) [file pgen.1009715.s013.docx]

**S3 Table.** Primer information for Gene Expression Assessed by qPCR analysis

| **Gene** | **Description** | **Sequence** | **Forward primer** | **Reverse primer** |
| --- | --- | --- | --- | --- |
| ***mlh-1*** | MLH (MutL Homolog) family | T28A8.7 | GATTTTCGAACGAAAGGAGATGGA | GGCTTGGAGATGTGACCG |
| ***mlh-1*** | MLH (MutL Homolog) family | T28A8.7 | GAGAAGACGATGATGTGGATT | GCGATTGTGATGAGTTGGCA |
| ***msh-2*** | MutS Homolog | H26D21.2 | CAGCCGATGAATACAGTATTTCT | CAGGAAGAGTCTTGCATTTGT |
| ***msh-2*** | MutS Homolog | H26D21.2 | ATTCACATGCTCTACAAGGT | TGCTATCGATGACAAGCTTCT |
| ***tba-1*** | alpha tubulin | F26E4.8 | CACTGATCTCTGCTGACAAGGCTTACC | TCTTGGTCTTGATGGCGGCG |
| ***tba-1*** | alpha tubulin | F26E4.8 |  |  |
| ***fcd-2*** | human FANCD2 ortholog | Y41E3.9 | GATGATGATGCGGAGACCAG | GAAGAATGTCACGAACTGCAC |
| ***fcd-2*** | human FANCD2 ortholog | Y41E3.9 | GAAATCGTGTCTTGCAGAG | CATTGCCGAATGAATTACTCG |
| ***fncm-1*** | Fanconi anemia complex component M homolog | F56A3.1 | CAAATCTTCAATTATCCGACTG | GAATTGTCTTCAGATTTCCACG |
| ***fncm-1*** | Fanconi anemia complex component M homolog | F56A3.1 | CTACTCGATGTACTTGTGTCAG | GAAAGTGGAAGACCATCGG |
| ***egl-1*** | DNA damage response marker | F23B12.9 | CTCCTCGTCTCAGGACTTCT | CGAAGTCATCGCACATTGCT |
| ***csb-1*** | ortholog of human ERCC6 | F53H4.1 | GAAAGTCAAGCAAGCCTTGGA | CATTTCGTCCGCCAATATTC |
| ***xpc-1*** | ortholog of human XPC . | Y76B12C.2 | TCAACCATCGGAACCACCG | TGTTTTCGGCTCGGTTTTGT |
| ***atm-1*** | Ortholog of human ATM serine/threonine kinase | Y48G1BL.2 | TCGAAGCGAGAGATTTCATTCTTC | GATCATGGCGAGCTTCACGA |
| ***atl-1*** | Ortholog of human ATR serine/threonine kinase | T06E4.3 | GTGCAGTTGAAAGAATGACATC | AGTCTTTAGAGAGATCGTCGTCT |
