# Supplementary material for: Histone demethylase AMX-1 is necessary for proper sensitivity to interstrand crosslink DNA damage
Source: PLoS Genet. 2021 Jul 30;17(7):e1009715. doi: 10.1371/journal.pgen.1009715 (PMC8357103; doi:10.1371/journal.pgen.1009715)
Supplement: Attachment — Submitted filename: Zhang et al Review_April 2021.pdf [file pgen.1009715.s014.pdf]

Review of Zhang et al., **Histone demethylase AMX-1 provides sensitivity to interstrand crosslink DNA damage.**

In this manuscript, the authors use the nematode *C. elegans* to investigate the role of AMX-1, a conserved yet poorly characterized lysine demethylase homologous to LSD2. As AMX-1 shares sequence similarity to the LSD1 homolog SPR-5, a known regulator of DNA repair, the authors' work tests the logical hypothesis that AMX-1 also mediates repair in *C. elegans*, using the germ line as a mode to test this. The primary finding in this paper is that AMX-1 plays a role in the repair of DNA crosslinks, which may be connected to epigenetic mechanisms in mitotic and meiotic germ cells. Additionally, the authors interrogate the relationship between AMX-1 and SPR-5, uncovering both redundant and non-redundant activities. Their data also uncover a novel role for AMX-1 in ICL repair that is independent from Fanconi Anemia activity.

Overall, this is a compelling and interesting story that was very well-written with rigorously designed experiments. The data were all very clearly depicted in the accompanying figures. In light of these strengths, the authors' findings would be bolstered by additional experiments that would and lead to a more comprehensive and mechanistic understanding of AMX-1 function. Below is a summary of critiques regarding data interpretation and suggestions for improving the narrative following by a list of minor critiques/suggestions.

1. The authors provide convincing evidence for nonredundant functions of AMX-1 and SPR-5 based on differences in H3K4me2 staining. Specifically, the differences in K4 staining in pre-meiotic nuclei of single mutants support the authors' assertion that AMX-1 plays a role in mitotic cells not shared by SPR-5. These data would be more convincing if H4K4me2 staining was also performed in *spr-5;amx-1* double mutants, an experiment that should be feasible given that the strain is viable as it was used for assessment of total H3K4me2 levels in Katz et al., 2009. I also would have liked to see this relationship (between AMX-1 and SPR-5) explored more thoroughly; for instance, are H3K4me2 levels higher in germline-specific western blots of *apx-1* and *spr-5;amx-1* double mutants vs. *spr-5*?

2. Though increased phosphoCHK-1 staining supports the authors' assumptions that loss of AMX-1 induces the DDR, these data alone do not rule out the possibility pCHK-1 staining is a consequence of other checkpoints being activated in *amx-1* mutants (e.g., the synapsis checkpoint). The possibility that other checkpoints are activated in *amx-1* mutants is indicated by the residual apoptotic nuclei observed in *amx-1;cep-1* double mutants. The authors conclusions would be more compelling if further experiments were conducted to tease out between these possibilities. For example, it would be interesting to know whether apoptosis is fully suppressed in

amx-1;cep-1;pch-2 (which would abrogate both the DDR and synapsis checkpoints) or whether pCHK-1 foci are still present in the *amx-1;cep-1* double mutants. Likewise, it would be useful to know whether pCHK-1 foci are detected in *amx-1* mutants deficient for upstream components of the DDR (e.g., ATM/ATR). The authors could also assess other markers of DDR activation by performing quantitative RT-PCR analysis of transcriptional targets of the DDR such as *egl-1*. (Note: I am not suggesting the authors performing *all* of these experiments, but it would make improve the manuscript to have a more in-depth understanding of how and why loss of AMX-1 leads to checkpoint activation.)

3. Since ICLs are an intermediate that when unrepaired produce DSBs and activate the DDR, it was not entirely clear, as written, how of AMX-1 could render the animals insensitive to nitrogen mustard yet still activate the DDR under wild-type conditions. Presumably, CHK1 is responding to endogenous sources of DNA damage in *amx-1* mutants without drug treatment, so would be useful to readers if the authors include a graphical model that discerns between these outcomes.

4. The conclusions that AMX-1 is highly sensitive to ICL-inducing agents would be stronger if the authors also examine agents other then HN2; it would be useful to determine whether insensitivity is also observed in animals treated with cisplatin.

5. From the authors' experiments testing RAD-51 foci, two key questions were left unanswered: (1) are these breaks *spo-11*-dependent and (2) are they observing a *delay* in repair or an *increase* in total break number? It would be helpful to address these possibilities by quantifying RAD-51 in *spo-11;amx-1* germ lines as well as in *rad-54;amx-1* germ lines.

6. The observation that RAD-51 foci are observed in *amx-1* mutants through late pachytene but are absent from diplotene suggest the possibility that persisting breaks may be repaired by NHEJ; it would be interesting to know whether this is the case, which could easily be addressed by quantifying RAD-51 in an *amx-1;lig-4* and/or *amx-1;cku-80* double mutants.

<u>Minor critiques</u>

1. Page 12: "We observed an increased number of gonads with gaps in amx-1 mutants." - Please more clearly explain this phenotype; readers unfamiliar with *C. elegans* germline biology may not understand the significance of this.

2. Page 12: "Consistent with the phenotypic results, AMX-1-GFP signal is mainly observed in the nuclei of gut cells…" As the aforementioned results only describe AMX-1's role in the germ line, the significance of it in being in the gut is not clear. It is also not explained in the Discussion. Since AMX-1 staining in the gut is also altered by *spr-5* mutants (page 13), this phenotype should be explained better.

3. Page 13: "No obvious signal was observed in control wild type supporting…" – phrasing is not clear as stated; it appears there is a word missing between wild-type and supporting. As a minor suggestion, I would also recommend the authors examine gfp expression in *amx-1* RNAi knockdown animals as a control.

4. Page 13: "Moreover, nuclei from premeiotic tip to diakinesis stages for the most part do not exhibit AMX-1-GFP signal, except for 5% of the premeiotic tip and pachytene nuclei where it was detected …" Is the 5% statistically significant? (Please indicate this in the text.)

5. Page 18: The language used to explain the results of the RAD-51 staining is inconsistent with the authors' statistical analysis. For example, authors refer to a "mild elevation" in RAD-51 in early pachytene, yet the p value is 0.8 compared to wild-type. Therefore, it is not accurate to state that RAD-51 is elevated.

6. Page 22: Please change "Similarly, *t*he lack of AMX-1" to "Similarly, the lack of AMX-1" (There is an italicized "t" that should not be italicized.)

7. Page 35: Please correct "error bards" to "error bars"

8. Page 38: Based on morphology, the panel representing *spr-5* labeled "premeiotic tip" appears to be pachytene nuclei; please check this. Additionally, is it possible to show a representative full-length germ line from each genotype stained with H3K4me2 in addition to or instead of the close-up panels?

9. Page 39: In Figure 5A, "premeiotic" is misspelled.

10. Page 39: In Figure 5B, "hydroxyurea" is misspelled.

11. Page 39: In Figure 5C, "premeiotic" is misspelled.

12. Page 43: In Figure 7C, there is a lot of background in the image of RAD-51 in the *amx-1* mutant, and the data are not representative of the actual number of RAD-51 foci quantified in the graph. Is the nucleus in the upper left of the *amx-1* panel apoptotic? If so, it might help to indicate this in the legend as it appears that there

are far greater then 10 foci. (It also looks like there is an apoptotic nucleus in the wild-type panel above.)
