# Supplementary material for: Histone demethylase AMX-1 is necessary for proper sensitivity to interstrand crosslink DNA damage
Source: PLoS Genet. 2021 Jul 30;17(7):e1009715. doi: 10.1371/journal.pgen.1009715 (PMC8357103; doi:10.1371/journal.pgen.1009715)
Supplement: Attachment — Submitted filename: Response to reviewers.docx [file pgen.1009715.s015.docx]

Dear Dr. Gregory P. Copenhaver and Gregory Barsh,

Thank you very much for allowing us the opportunity to submit the revision of ‘Histone demethylase AMX-1 is necessary for proper sensitivity to interstrand crosslink DNA damage’ for publication in *PLoS* *Genetics*. We highly appreciate the time and effort you and the reviewers dedicated to providing feedback on our manuscript. We are grateful for the insightful comments and valuable improvements to our manuscript. We incorporated most of the suggestions made by the reviewers. The revised text and additional experiments further strengthen our original findings. We hope that the corrections will meet with your approval for this journal. All page numbers refer to the revised manuscript file with tracked changes. The responses to the reviewer’s comments are highlighted in blue below:

**Comments to the Authors:
Reviewer #1** The manuscript by Zhang et al., describes the characterization of the demethylase AMX-1 in C. elegans. The findings have the potential to provide insight into the role of chromatin in DNA damage repair and ultimately how chromatin changes contribute to dysregulation in disease states. The authors find that this demethylase is primarily expressed in mitotic cells but plays a role in the germ line and promotes sensitivity to ICLs. Interestingly, the protein appears to be regulated by ubiquitination and in turn regulates the expression of the mismatch repair MutL. Overall, there is some interesting findings in this manuscript but significant rewriting and incorporation of new experiments would be important to make a compelling case for the role of this demethylase in DNA damage repair.
1) The authors describe AMX-1 as “uncharacterized” and this as the first report of this demethylase being involved in ICL repair. While it is true that this is the first report in ICL repair (I think), the study by Wang et al., 2020 (Nat Struct Mol Biol) is related and the authors need to consider these published findings in this paper. Wang et al. show that the amx-1 mutant is resistant to UV (L1 based assay) and the evidence suggests that H3K4me2 levels are important for recovery from DNA damage due to global gene expression changes. Incorporating this paper will require a number of changes throughout the manuscript.

We greatly appreciate the reviewer’s comment. We agree with the reviewer’s assessment and incorporated multiple changes in the text as requested. Now it reads as follows:

Introduction Page 4) H3K4me2 levels are important for recovery from DNA damage repair due to global gene expression changes (1).

Results Page 12) To test this, we examined whether AMX-1 regulates the proper expression of genes in the Fanconi anemia (FA), mismatch repair, and nucleotide excision repair (NER) pathways, all of which have been implicated in ICL repair (1-6). While mRNA expression of two key FA components, *fcd-2* and *fncm-1*, and two key NER components, *csb-1* and *xpc-1*, was not significantly altered, interestingly, *amx-1* mutants exhibited an ~43% reduction in *mlh-1* expression compared to wild type (Fig 5D)

Discussion Page 18) Of note, a recent study by Wang *et al*. reported that the *amx-1* mutant is resistant to UV irradiation and UV-induced H3K4me2 is independent of nucleotide excision repair (1). While this may seem to be contradictory to our result (Fig 5B), our assay exposes adult worms to UVC to measure levels of hatching (embryonic viability) and therefore is focused on effects taking place within the germline and/or embryonic development. Wang *et al*. monitored purely larval growth, which could account for the different DNA damage sensitivity outcomes.

Wang et al. analyzed larval growth while we analyzed embryonic lethality. Thus, we mainly monitor nuclei development from germline to embryos while their study focused on mitotic growth. Since the two reports focus on different stages and events (embryonic hatching vs. larval development), these two reports do not contradict each other.

2) I recommend changing the title – AMX-1 doesn’t “provide” sensitivity.

Thank you for pointing this out. We have revised the manuscript and now the title reads as follows:

Title) Histone demethylase AMX-1 is necessary for proper sensitivity to interstrand crosslink DNA damage

Additionally, in the author summary: “how defective histone demethylase contributes to DNA damage repair” – please rephrase with respect to the wild-type function.

Thank you for pointing this out. We have revised the manuscript and now it reads:

Page 3) however, the mechanisms by which histone demethylases contribute to DNA damage repair are not very clear.

3) Figure 2: Please change the label of the Y axis of left most graph of panel A. It is currently “Larval arrest (%adult)” this is confusing. The other graphs show the defect – on this one it is the wild-type phenotype, although the label indicates the mutant phenotype.

Thank you very much for this correction. We have revised the manuscript and now it reads:

Figure 2A) Adult (%)

4) Figures 3 and 4: Could the GFP signal in the gut and sheath cells be enhanced? It is very difficult to see. I am also struggling with the lack of expression in the germ line, yet the effect on H3K4me2 is evident in germ cells. It would be really nice to examine tissue-specific inactivation of AMX-1 to determine whether it functions cell autonomously/non-autonomously.

Thank you for this suggestion. Per the reviewer’s request, GFP signals in the gut and sheath cells have been enhanced. Although we agree that tissue-specific inactivation would be nice to examine, please consider that it is a little beyond the scope and we stayed focused on DNA damage repair function primarily in the germline in this study.

5) Figure 5: The CHK-1 signal is not very convincing – this is particularly true for the "premeiotic (misspelled) tip-TZ" as many of the foci in the amx-1 mutant do not overlap with the DAPI signal; new images and quantification need to be included. Please also quantify the “chromosome fragments” observed in the presence of crosslinking agents. I find it confusing why you see elevation of chromosome fragments but a resistance to ICLs in the mutant. This needs to be discussed. In terms of the lack of effect of UV – this will need to be addressed here given the previous publication. The authors also show that there is a reduction in mlh-1 expression in amx-1. I recommend that the authors also look at the expression of NER genes, as NER plays an important role in ICL repair in C. elegans.

Thank you very much for these suggestions. We performed new pCHK-1 immunostaining and revised the manuscript accordingly. In the new images of pCHK-1 (Figure 5), most foci overlap with the DAPI signal. Importantly, this antibody has been used for other report and exhibited some portion of foci not overlapping with the DAPI signal (7).

Also, per the reviewer’s request, we quantified the chromosome fragments and also discussed the data and not it reads:

Page 33) 6 out of 30 (20%) gonads contained broken chromosomes for amx-1 and 0 out of 20 (0%) gonads for wild type.

Page 19) It is worth noting that although the amx-1 mutants bear tolerance to ICL damage, they also display chromosome fragmentation at the premeiotic tip (Fig 5C), suggesting that loss of AMX-1 may result in multiple phenotypes not only restricted to defects in ICL repair. Consistent with this, amx-1 mutants exhibited both CEP-1/p53-dependent and -independent apoptosis (Fig 7E) as well as embryonic lethality, reduced brood size, altered germ cell progression and DNA damage checkpoint activation.

Per the reviewer’s request, we explained the lack of UVC effects. Now it reads as follows:

Page 18) Of note, a recent study by Wang et al. reported that the amx-1 mutant is resistant to UV irradiation and UV-induced H3K4me2 is independent of nucleotide excision repair (12). While this may seem to be contradictory to our result (Fig 5B), our assay exposes adult worms to UVC to measure levels of hatching (embryonic viability) and therefore is focused on effects taking place within the germline and/or embryonic development. Wang et al. monitored purely larval growth, which could account for the different DNA damage sensitivity outcomes.

Per the reviewer’s request, we analyzed the expression of NER genes (Figure 5D). Now it reads:

Page 12) To test this, we examined whether AMX-1 regulates the proper expression of genes in the Fanconi anemia (FA), mismatch repair, and nucleotide excision repair (NER) pathways, all of which have been implicated in ICL repair (12, 13, 27-30). While mRNA expression of two key FA components, *fcd-2* and *fncm-1*, and two key NER components, *csb-1* and *xpc-1*, was not significantly altered, interestingly, *amx-1* mutants exhibited an ~43% reduction in *mlh-1* expression compared to wild type (Fig 5D).

6) The authors argue that AMX-1 expression compensates for lack of another demethylase, SPR-5. I think it will be important to examine the phenotypes of the amx-1; spr-5 double mutant compared to each of the single mutants to say more about the role of these demethylases in repair and whether or not they compensate for each other. As part of this, is there a progressive sterility phenotype of amx-1 as there is with spr-5?

We highly appreciate the reviewer’s comments. Regarding progressive sterility, in contrast to *spr-5,* no distinct progressive sterility was observed in either early (F2-10) or late generations (>F20) of *amx-1* mutants.

In terms of phenotypes, we compared HN2 and IR sensitivity among *spr-5, amx-1* and *spr-5;amx-1* and found that SPR-5 and AMX-1 function separately in DNA damage repair. Thus, we believe SPR-5 and AMX-1 have independent roles in DNA damage repair. Of note, we are preparing a new manuscript where we mainly focus on the fertility/sterility of the two.

To further address the reviewer’s question, we performed H3K4me2 staining in the *spr-5;amx-1* double mutants. The double mutants did not exhibit additive or synergistic changes in histone methylation level compared to *amx-1* single mutants (Figure 4). This now reads as follows:

Figure 4 and Page 9) Consistent with this, *spr-5;amx-1* double mutants did not display additive or synergistic histone methylation levels relative to *amx-1* single mutants (in premeiotic tip nuclei, P=0.1461; in embryos, P=0.3080; in pachytene nuclei, P=0.6434; and in gut cells, P=0.7680).

7) The way Figure 7 is set up, suggests that you are examining RAD-51 following treatment of ICLs, but you are not. Please rewrite the set up for this experiment. The authors may want to consider examining DAPI-staining bodies in the cep-1; amx-1 double mutant to determine whether there are aberrant nuclei that are culled by apoptosis.

We agree with the reviewer and changed the manuscript per the reviewer’s request. Now it reads:

Page 13) Nuclei are positioned in a temporal-spatial manner along the germline in *C. elegans*, proceeding in a distal to proximal orientation from mitosis into the various stages of meiotic prophase I. Taking advantage of this well-defined organization, levels of RAD-51 foci, which mark sites undergoing DSB repair (32), were quantitated both in mitotic (premeiotic tip) and meiotic nuclei (transition zone and pachytene) and compared between wild type and *amx-1* mutants (Fig 7A).

Also, per the reviewer’s request, we examined the number of chromosome fragments in the *cep-1;amx-1* mutants and found that the majority of fragments were no longer detected. Now it reads:

Page 15) Consistently, DAPI-stained fragments observed in premeiotic tip nuclei upon HN2 exposure (Fig 5C) were also rescued in *cep-1;amx-1* mutants (6.7% gonads contain fragments vs. 20% in *amx-1*).

**Reviewer #2:**  In this study, the authors claim that lysine-specific histone demethylase 2 (LSD2) homolog, AMX-1 provides sensitivity to DNA inter-strand crosslinks. They showed resistance against nitrogen mustard (HN2) which cause DNA inter-strand crosslinks in amx-1 mutants compared with wild-type. The mRNA expression of mismatch repair protein MLH-1 decreased in amx-1 mutants. This is reminiscent to the loss of MLH1 expression induces the resistance to DNA intra-strand crosslink caused by cisplatin in the human cancer cell line. Histone H3 K4me2 accumulates in amx-1 mutants suggesting that AMX-1 is required for demethylation of H3K4me2. While LSD2 harbors RING-like zinc finger motif and acts as an E3 ubiquitin ligase, AMX-1 does not have the zinc finger motif. But still they showed ubiquitination-dependent relocalization of AMX-1::GFP in embryos after treatment of HN2. RAD-51 foci accumulate in amx-1 mutants however they could not reach the conclusion that those are accumulations of repair intermediates or hyper activation of DNA repair machinery.
We highly appreciate the reviewer’s comments.

**Minor Comments**
p.19-20,
In the strain and alleles section, his6 follows after amx-1::gfp. In the transgenic worms section, the authors described that GFP-HA is inserted before the stop codon of amx-1 gene. Which is correct his6 and HA?

Thank you for pointing this out. We changed the text accordingly (It is GFP-HA). Now it reads:

Page 22) *amx-1(rj20[amx-1::gfp::ha + loxP unc-119(+) loxP])*

p.17 line6	the -> the

Thank you for pointing this out. We changed the text accordingly.

Page 19) Similarly, the lack of AMX-1

**Major Comments**

p.31 Fig3, p.36 Fig6
The number of cells in the embryos is not matched in WT and spr-5 mutants at Fig3A. Similarly, the left embryo at the right panels in the amx-1::GFP+HN2 in Fig 6A looks comma stage. The stages of embryogenesis should be matched between controls and samples though the paper.

Thank you for pointing this out. Per the reviewer’s request, we matched the number of cells in the embryos of WT and *spr-5* mutants in Figure 3A. Also, we matched the stage of embryogenesis scored for *amx-1*::GFP+HN2 in Figure 6A.

p.34 Fig5B
I suggest that the authors try to add additional doses in both camptothecin and Hydroxyurea because there are no decreases of hatching of wild-type in 500nM of CPT and 15mM HU even if some degree of decrease were observed in positive control mutants (him-18 and ztf-8 respectively).

We agree with the reviewer and have added additional doses in both camptothecin and hydroxyurea and this data can be seen in Figure 5B. In brief, consistent with our prior results, higher doses of both camptothecin and HU did not alter hatching (%) between *amx-1* and wild type significantly. Now it reads:

Page 11) In addition, UVC, which induces cyclobutane pyrimidine dimers, and camptothecin, which results in a single-ended DNA DSB when collision of a replication fork occurs at the lesion, did not reduce hatching levels in *amx-1* mutants compared to wild type (Fig 5B, for UVC, 99% (wt) vs 100% (*amx-1*) at 100 J/m^2^, P=0.166,1 and 97% (wt) vs 96% (*amx-1*) at 200 J/m^2^, P=0.6858; for camptothecin, 98% (wt) vs 99% (*amx-1*) at 100 nM, P=0.4038, 97% (wt) vs 96% (*amx-1*) at 500 nM, P=0.1374, and 92% (wt) vs 93% (*amx-1*) at 750 nM, P=0.5669). Exposure to hydroxyurea (HU), which results in a checkpoint-dependent cell cycle arrest, led to no significant changes in hatching in *amx-1* mutants compared to wild type (99% and 99%, respectively, at 15 mM, P=0.6538, and 93% (wt) vs 97% (*amx-1*) at 25 mM, P= 0.2228) while lack of ZTF-8, an ortholog of RHINO, induced sensitivity as previously shown (22).

p.10
I agree that some degrees of NH2 resistance were observed in amx-1 mutants. If you claim this was caused by DNA inter-strand crosslinks, it worth to check to use other reagents causing DNA inter-strand crosslinks, such as mitomycin C. And if the authors want to compare the phenomenon in the cancer cell line in which the expression of MLH1 decreased and C. elegans amx-1 mutants, intra-strand crosslinks inducing reagents, such as cisplatin should be used in the series of DNA damage sensitivity assay.

Thank you for pointing this out. Per the reviewer’s suggestion we extended this analysis and used cisplatin. We found that the *amx-1* mutant is also tolerant to cisplatin further validating the tolerance to ICLs exhibited in *amx-1* mutants. This result has been incorporated into Figure 5B. Now it reads:

Page 10-11) Consistently, *amx-1* mutants also exhibited tolerance to cisplatin, which produces DNA crosslinks (83% vs 92% at 300 µM P=0.0119).

P.13
If the authors argue that there is HN2 resistance in amx-1 mutants, the levels **of RAD-51 foci** provably decrease in amx-1 mutants compared with wild-type after treatment of HN2 if RAD-51 foci indicate the accumulation of DNA damages. This particular experiment will be required to prove the resistance. **Observe RAD-51 foci and AMX-1::GFP** localization in the embryo after treatment of HN2.

Thank you for pointing this out. We analyzed RAD-51 foci levels after HN2 treatment and found that these were decreased in *amx-1* mutants compared to wild type. This data has been incorporated to the manuscript (Figure 7D) and now it reads:

Page 14) Finally, since *amx-1* mutants exhibit tolerance against HN2, it is plausible that RAD-51 foci levels might decrease in *amx-1* mutants compared with wild-type after HN2 treatment. Indeed, wild type worms exhibited gonads with higher levels of RAD-51 compared to *amx-1* mutants, further validating the ICL tolerance (Fig 7D). Of note, no distinct RAD-51 foci are observed in wild type control embryos after HN2 exposure (Supplemental Fig 8).

Per the reviewer’s request, we also performed RAD-51 and AMX-1::GFP dual immunostaining in the embryos after HN2 treatment (Supplemental Figure 8). No distinct RAD-51 foci were observed while AMX-1::GFP signal was detected. Now it reads:

Supplemental figure 8**.** Co-immunostaining of RAD-51 and AMX-1::GFP signals of early embryos treated with HN2. No distinct RAD-51 foci were observed in embryos in wild type control. *amx-1*::GFP animals were treated with 150 µM of HN2 in NGM containing *E. coli* OP50 for 20 hours. Inset as higher magnification images. Bar= 2 μm.

p.17
It is also worth to check whether **HN2 (and cisplatin) resistance is observed in mlh-1 mutants.**

Per the reviewer’s request, we examined cisplatin resistance upon *mlh-1* depletion and found that *mlh-1* (RNAi) worms exhibited cisplatin and nitrogen mustard resistance. The text has been modified accordingly and now it reads:

Page 12) To explore whether proper expression of MLH-1 allows susceptibility to ICLs, we depleted MLH-1 expression by RNAi and assessed the frequency of hatching upon exposure to DNA crosslinking damage by either HN2 or cisplatin. Lack of MLH-1 expression resulted in increased tolerance to both HN2 and cisplatin compared to control, indicating that down-regulation of MLH-1 may be sufficient for the ICL tolerance displayed by *amx-1* mutants (Supplemental Fig 7).

**Supplemental Fig 7.** Relative hatching observed for eggs laid by either control worms (empty vector; pL4440) or worms depleted of *mlh-1* by RNAi after treatment with the indicated doses of nitrogen mustard and cisplatin. 8% vs 24% at 150 μM HN2, P= 0.0003, and 82% vs 95% at 300 μg/ml cisplatin, P<0.0001, by the two-tailed Mann-Whitney test, 95% C.I.

**Reviewer #3**: review is uploaded as an attachment

In this manuscript, the authors use the nematode C. elegans to investigate the role of AMX1, a conserved yet poorly characterized lysine demethylase homologous to LSD2. As AMX-1 shares sequence similarity to the LSD1 homolog SPR-5, a known regulator of DNA repair, the authors’ work tests the logical hypothesis that AMX-1 also mediates repair in C. elegans, using the germ line as a mode to test this. The primary finding in this paper is that AMX-1 plays a role in the repair of DNA crosslinks, which may be connected to epigenetic mechanisms in mitotic and meiotic germ cells. Additionally, the authors interrogate the relationship between AMX-1 and SPR-5, uncovering both redundant and non-redundant activities. Their data also uncover a novel role for AMX-1 in ICL repair that is independent from Fanconi Anemia activity.

Overall, this is a compelling and interesting story that was very well-written with rigorously designed experiments. The data were all very clearly depicted in the accompanying figures. In light of these strengths, the authors¡¯ findings would be bolstered by additional experiments that would and lead to a more comprehensive and mechanistic understanding of AMX-1 function. Below is a summary of critiques regarding data interpretation and suggestions for improving the narrative following by a list of minor critiques/suggestions. 1. The authors provide convincing evidence for nonredundant functions of AMX-1 and SPR-5 based on differences in H3K4me2 staining. Specifically, the differences in K4 staining in pre-meiotic nuclei of single mutants support the authors¡¯ assertion that AMX-1 plays a role in mitotic cells not shared by SPR-5. These data would be more convincing if H4K4me2 staining was also performed in *spr-5;amx-1* double mutants, an experiment that should be feasible given that the strain is viable as it was used for assessment of total H3K4me2 levels in Katz et al., 2009. I also would have liked to see this relationship (between AMX-1 and SPR-5) explored more thoroughly; for instance, are H3K4me2 levels higher in germline-specific western blots of *apx-1* and *spr-5;amx-1* double mutants vs. *spr-5*?

We greatly appreciate the reviewer’s comment. We performed the H3K4me2 staining using the *spr-5;amx*-1 double mutants. Double mutants appeared unaltered compared to amx-1 single mutants (Figure 4), supporting distinct roles for the two genes. Now it reads:

Figure 4 and Page9) Consistent with this, *spr-5;amx-1* double mutants did not display additive or synergistic histone methylation levels relative to *amx-1* single mutants (in premeiotic tip nuclei, P=0.1461; in embryos, P=0.3080; in pachytene nuclei, P=0.6434; and in gut cells, P=0.7680).

Unfortunately, we were not able to perform germline-specific western blots due to a lack of personnel in the lab (access to the labs has been limited and we had to prioritize what could be done in a reasonable amount of time). However, we compared HN2 and IR sensitivity between single and double mutants (*spr-5, amx-1* and *spr-5;amx-1*) and found that SPR-5 and AMX-1 function separately in DNA damage repair; these proteins function in parallel. Thus, we believe SPR-5 and AMX-1’s roles in DNA damage repair are independent. Also the progressive sterility exhibited in *spr-5* was not observed in *amx-1* mutants. Of note, we are preparing a new manuscript where we mainly focus on the fertility/sterility of these two.

2. Though increased phosphoCHK-1 staining supports the authors¡¯ assumptions that loss of AMX-1 induces the DDR, these data alone do not rule out the possibility pCHK-1 staining is a consequence of other checkpoints being activated in *amx-1* mutants (e.g., the synapsis checkpoint). The possibility that other checkpoints are activated in *amx-1* mutants is indicated by the residual apoptotic nuclei observed in *amx-1;cep-1* double mutants. The authors conclusions would be more compelling if further experiments were conducted to tease out between these possibilities. For example, it would be interesting to know whether apoptosis is fully suppressed in *amx-1;cep-1;pch-2* (which would abrogate both the DDR and synapsis checkpoints) or whether pCHK-1 foci are still present in the *amx-1;cep-1* double mutants.

Thank you very much for pointing this out. We performed the experiment per the reviewer’s suggestion and found that pCHK-1 levels in *cep-1;amx-1* are higher than in *cep-1* (Fig 7E). The manuscript has been changed accordingly.

Page 16) The higher level of pCHK-1 foci in the double mutant also supports this idea (Fig 7E).

Page 35) Right, Quantification of pCHK-1 foci in pachytene nuclei in the indicated genotypes. P=0.0299 for *cep-1;amx-1* and *cep-1* by the t-test, 95% C.I.

Likewise, it would be useful to know whether pCHK-1 foci are detected in *amx-1* mutants deficient for upstream components of the DDR (e.g., ATM/ATR).

We performed the experiment per the reviewer’s suggestion and found that the increased levels of pCHK-1 foci detected in *amx-1* mutants requires upstream components of the DDR pathway since they were no longer observed in the *atl-1*(RNAi) knockdown background (Figure 5A). Now it reads:

Figure 5A and Page 10) Increased pCHK-1 signal is detected in embryos, premeiotic tip-transition zone and pachytene nuclei and this increase requires the upstream pathway component ATL-1 (*C. elegans* ATR homolog).

The authors could also assess other markers of DDR activation by performing quantitative RT-PCR analysis of transcriptional targets of the DDR such as *egl-1*. (Note: I am not suggesting the authors performing *all* of these experiments, but it would make improve the manuscript to have a more in-depth understanding of how and why loss of AMX-1 leads to checkpoint activation.)

Thank you for pointing this out. We performed qRT-PCR per the reviewer’s suggestion and found an increased level of *egl-1* mRNA expression in *amx-1* mutants, consistent with the elevated pCHK-1 signal observed in this background. This new result is incorporated into Figure 5A and now reads:

Figure 5A and Page10) Consistently, *amx-1* mutants exhibit a higher level of *egl-1* expression, a DNA damage response marker, suggesting the lack of AMX-1 expression activates the DNA damage checkpoint in an ATL-1 dependent manner.

3. Since ICLs are an intermediate that when unrepaired produce DSBs and activate the DDR, it was not entirely clear, as written, how of AMX-1 could render the animals insensitive to nitrogen mustard yet still activate the DDR under wild-type conditions. Presumably, CHK1 is responding to endogenous sources of DNA damage in *amx-1* mutants without drug treatment, so would be useful to readers if the authors include a graphical model that discerns between these outcomes.

Thank you very much for pointing this out. We agree with the reviewer. Thus, we have included a model figure paying attention to the absence and presence of HN2-induced damage (Figure 8). Now it reads:

Page 35) Fig 8. Model for the role of histone demethylase AMX-1 in DNA damage repair

Top, in wild-type animals, we propose that AMX-1 and SPR-5 act in both redundant and non-redundant manners. Upon ICL damage, AMX-1 is ubiquitylated and regulates the proper expression of MLH-1 (mismatch repair MutL homolog). Bottom, in the absence of AMX-1, SPR-5 expression compensates for the lack of AMX-1. A DNA damage checkpoint is activated in *amx-1* mutants in the absence of exogenous damage, suggesting that lack of AMX-1 responds to endogenous DNA damage. Upon HN2 exposure, lack of AMX-1 leads to reduced MLH-1 expression and ICL tolerance.

4. The conclusions that AMX-1 is highly sensitive to ICL-inducing agents would be stronger if the authors also examine agents other then HN2; it would be useful to determine whether insensitivity is also observed in animals treated with cisplatin.

Per the reviewer’s request, we performed cisplatin experiments and found that *amx-1* mutants are also tolerant to cisplatin. This new result has been incorporated into Figure 5B. It now reads:

Page 10-11) Consistently, *amx-1* mutants also exhibited tolerance to cisplatin, which produces DNA crosslinks (83% vs 92% at 300 µM P=0.0119).

5. From the authors¡¯ experiments testing RAD-51 foci, two key questions were left unanswered: (1) are these breaks *spo-11*-dependent and (2) are they observing a *delay* in repair or an *increase* in total break number? It would be helpful to address these possibilities by quantifying RAD-51 in *spo-11;amx-1* germ lines as well as in *rad-54;amx-1* germ lines.

We performed the *amx-1;spo-11* experiments per the reviewer’s request and changed the text accordingly and it now reads:

Page 14) To better understand DSB formation in *amx-1* mutants, we monitored the levels of RAD-51 foci in the germlines of *amx-1;spo-11* double mutants which lack the formation of programmed meiotic DSBs (Fig 7B). Elevated levels of RAD-51 foci in both mitotic (premeiotic tip; PMT) and meiotic (pachytene) nuclei in *amx-1;spo-11* double mutants compared to *spo-11* single mutants suggest these are SPO-11-independent RAD-51 foci. However, when we subtracted the mitotic DSBs found in *amx-1;spo-11* from the total number of RAD-51 foci in *amx-1* mutants, higher levels of RAD-51 were detected in mid and late pachytene in *amx-1* mutants suggesting that SPO-11-dependent meiotic DSBs are also elevated in *amx-1* mutants.

Regarding *rad-54*, we failed to obtain the correct *rad-54* mutants or RNAi clones promptly in China, thus not completing experiments at the moment. I hope you understand the pandemic condition in which we have difficulties getting worms from outside the U.S.

6. The observation that RAD-51 foci are observed in *amx-1* mutants through late pachytene but are absent from diplotene suggest the possibility that persisting breaks may be repaired by NHEJ; it would be interesting to know whether this is the case, which could easily be addressed by quantifying RAD-51 in an *amx-1;lig-4* and/or *amx-1;cku-80* double mutants.

Thank you for pointing this out. We per the reviewer’s request we examined amx-1 worms depleted of CKU-80 by RNAi. In short, *cku-80* depletion did not alter the levels of RAD-51 foci significantly compared to amx-1 single mutants. We changed the text accordingly and it now reads:

Page 14) However, repair of the elevated levels of RAD-51 foci detected in late pachytene may not be dependent on non-homologous end-joining since CKU-80 depletion in *amx-1* mutants did not result in elevated levels of RAD-51 foci persisting into late prophase I (Supplemental Fig 9).

Supplemental Fig 9). Quantitation of RAD-51 foci in germlines of the indicated genotypes. Mean numbers of RAD-51 foci observed per nucleus on each zone along the germline axis (x-axis). P= 0.8888 in *amx-1* and *amx-1 + cku-80* (RNAi) in early pachytene. P= 0.1763 in *amx-1* and *amx-1 + cku-80* (RNAi) in mid pachytene. P= 0.5509 in *amx-1* and *amx-1 + cku-80* (RNAi) in late pachytene. P= 0.2431 in *amx-1* and *amx-1 + cku-80* (RNAi) in diplotene by the two-tailed Mann–Whitney test, 95% C.I. Error bars represent standard error of the mean.

**Minor critiques**

1. Page 12: We observed an increased number of gonads with gaps in amx-1 mutants.¡± - Please more clearly explain this phenotype; readers unfamiliar with *C. elegans* germline biology may not understand the significance of this.

Thank you for pointing this out. We changed the text accordingly and now the manuscript reads:

Page 7) Furthermore, we observed an increased number of gonads with gaps in *amx-1* mutants while nuclei were organized in a normal temporal and spatial manner in wild type gonads.

2. Page 12: Consistent with the phenotypic results, AMX-1-GFP signal is mainly observed in the nuclei of gut cells As the aforementioned results only describe AMX-1’s role in the germ line, the significance of it in being in the gut is not clear. It is also not explained in the Discussion. Since AMX-1 staining in the gut is also altered by *spr-5* mutants (page 13), this phenotype should be explained better.

Thank you very much for pointing this out. We changed the text accordingly (results and discussion) and now the manuscript reads:

Page 8) however, no evident induction from premeiotic tip to diakinesis or mitotic sheath cells was observed. These observations suggest tissue-specific compensation involving the two histone demethylase genes.

Page 17) Interestingly, AMX-1::GFP signal was detected in gut cells (Fig 3). Studies in other species have identified roles for histone methylation in maintaining intestine integrity and proper immune response (38, 39). Given that AMX-1 is expressed in the gut cells and further altered by lack of SPR-5, the abovementioned functions may also exist in *C. elegans.*

3. Page 13: No obvious signal was observed in control wild type supporting –phrasing is not clear as stated; it appears there is a word missing between wild-type and supporting. As a minor suggestion, I would also recommend the authors examine gfp expression in *amx-1* RNAi knockdown animals as a control.

Thank you for pointing this out. We changed the text accordingly. Also we confirmed that *amx-1* RNAi resulted in loss of GFP signal (Supplemental Fig 4). Now it reads:

Page 8) No obvious signal was observed in the control (N2) strain or upon *amx-1* RNAi depletion, which supports the specificity of the antigen-antibody reaction (Fig 3A and Supplemental Fig 4).

4. Page 13: Moreover, nuclei from premeiotic tip to diakinesis stages for the most part do not exhibit AMX-1-GFP signal, except for 5% of the premeiotic tip and pachytene nuclei where it was detected ¡ Is the 5% statistically significant? (Please indicate this in the text.)

It is statistically significant and we changed the text accordingly. Now it reads:

Supplemental Fig 5. AMX-1 is rarely localized in the germline. We observed 5% of gonads containing AMX-1::GFP signal either in the premeiotic tip or pachytene stage (5 out of 100 and 5 out of 96, respectively). Wild type (N2) is used as a control. P=0.032 in control versus AMX-1::GFP in the premeiotic tip, and P=0.0248 in control versus AMX-1::GFP in pachytene, by the two-tailed t-test. Bars=10μm.

5. Page 18: The language used to explain the results of the RAD-51 staining is inconsistent with the authors¡¯ statistical analysis. For example, authors refer to a mild elevation in RAD-51 in early pachytene, yet the p value is 0.8 compared to wild-type. Therefore, it is not accurate to state that RAD-51 is elevated.

Thank you for pointing this out. We changed the text accordingly and now it reads:

Page 13) In *amx-1* mutants, the mildly elevated levels of RAD-51 foci observed at the premeiotic tip were not significantly different compared to wild type (Fig 7B, an average of 0.1 and 0.2 RAD-51 foci/nucleus for wild type and *amx-1*, respectively).

6. Page 22: Please change Similarly, *t*he lack of AMX-1 to Similarly, the lack of AMX1 (There is an italicized t that should not be italicized.)

Thank you for pointing this out. We changed the text accordingly.

Page 19) Similarly, the lack of AMX-1

7. Page 35: Please correct error bards to error bars

Thank you for pointing this out. We changed the text accordingly (Figure 2)

8. Page 38: Based on morphology, the panel representing *spr-5* labeled premeiotic tip¡± appears to be pachytene nuclei; please check this. Additionally, is it possible to show a representative full-length germ line from each genotype stained with H3K4me2 in addition to or instead of the close-up panels?

Thank you for pointing this out. We replaced the image to represent premeiotic nuclei better (Figure 4).

Also, we have created a new supplemental figure showing zoom-out images (Supplemental figure 10). Please note that we could not picture a whole germline with fluorescent imaging due to a technical limitation at the moment but rather photograph a small zone with 90-100x magnification as in Supplemental figure 10.

9. Page 39: In Figure 5A, premeiotic is misspelled.

(Figure 5A) Thank you for pointing this out. We changed the label in the figure accordingly.

10. Page 39: In Figure 5B, hydroxyurea is misspelled.

(Figure 5B) Thank you for pointing this out. We changed the label in the figure accordingly.

11. Page 39: In Figure 5C, premeiotic is misspelled.

(Figure 5C) Thank you for pointing this out. We changed the label in the figure accordingly.

12. Page 43: In Figure 7C, there is a lot of background in the image of RAD-51 in the *amx-1* mutant, and the data are not representative of the actual number of RAD-51 foci quantified in the graph. Is the nucleus in the upper left of the *amx-1* panel apoptotic? If so, it might help to indicate this in the legend as it appears that there are far greater then 10 foci. (It also looks like there is an apoptotic nucleus in the wild-type panel above.)

Thank you for pointing this out. We agree with the reviewer and changed the text accordingly and now it reads:

Page35 Figure 7C) *amx-1* mutants contain pachytene nuclei with >10 RAD-51 foci per nucleus which may undergo apoptosis (indicated by yellow arrows, 10 out of 25 gonads).

1.	Wang S, Meyer DH, Schumacher B. H3K4me2 regulates the recovery of protein biosynthesis and homeostasis following DNA damage. Nature structural & molecular biology. 2020;27(12):1165-77.

2.	Kim HM, Beese-Sims SE, Colaiacovo MP. Fanconi Anemia FANCM/FNCM-1 and FANCD2/FCD-2 Are Required for Maintaining Histone Methylation Levels and Interact with the Histone Demethylase LSD1/SPR-5 in Caenorhabditis elegans. Genetics. 2018;209(2):409-23.

3.	Niraj J, Farkkila A, D'Andrea AD. The Fanconi Anemia Pathway in Cancer. Annual review of cancer biology. 2019;3:457-78.

4.	Aebi S, Kurdi-Haidar B, Gordon R, Cenni B, Zheng H, Fink D, et al. Loss of DNA mismatch repair in acquired resistance to cisplatin. Cancer research. 1996;56(13):3087-90.

5.	Wilson DM, 3rd, Rieckher M, Williams AB, Schumacher B. Systematic analysis of DNA crosslink repair pathways during development and aging in Caenorhabditis elegans. Nucleic acids research. 2017;45(16):9467-80.

6.	Oh S, Bae W, Alfhili MA, Lee MH. Nucleotide Excision Repair, XPA-1, and the Translesion Synthesis Complex, POLZ-1 and REV-1, Are Critical for Interstrand Cross-Link Repair in Caenorhabditis elegans Germ Cells. Biochemistry. 2020;59(38):3554-61.

7.	Bailly AP, Freeman A, Hall J, Declais AC, Alpi A, Lilley DM, et al. The Caenorhabditis elegans homolog of Gen1/Yen1 resolvases links DNA damage signaling to DNA double-strand break repair. PLoS Genet. 2010;6(7):e1001025.

8.	Long DT, Raschle M, Joukov V, Walter JC. Mechanism of RAD51-dependent DNA interstrand cross-link repair. Science. 2011;333(6038):84-7.

9.	Colaiacovo MP, MacQueen AJ, Martinez-Perez E, McDonald K, Adamo A, La Volpe A, et al. Synaptonemal complex assembly in C. elegans is dispensable for loading strand-exchange proteins but critical for proper completion of recombination. Dev Cell. 2003;5(3):463-74.

10.	Kim HM, Colaiacovo MP. ZTF-8 Interacts with the 9-1-1 Complex and Is Required for DNA Damage Response and Double-Strand Break Repair in the C. elegans Germline. PLoS Genet. 2014;10(10):e1004723.

11.	Dupret B, Volkel P, Vennin C, Toillon RA, Le Bourhis X, Angrand PO. The histone lysine methyltransferase Ezh2 is required for maintenance of the intestine integrity and for caudal fin regeneration in zebrafish. Biochimica et biophysica acta Gene regulatory mechanisms. 2017;1860(10):1079-93.

12.	Angrisano T, Pero R, Peluso S, Keller S, Sacchetti S, Bruni CB, et al. LPS-induced IL-8 activation in human intestinal epithelial cells is accompanied by specific histone H3 acetylation and methylation changes. BMC microbiology. 2010;10:172.
