# Supplementary material for: Histone demethylase AMX-1 is necessary for proper sensitivity to interstrand crosslink DNA damage
Source: PLoS Genet. 2021 Jul 30;17(7):e1009715. doi: 10.1371/journal.pgen.1009715 (PMC8357103; doi:10.1371/journal.pgen.1009715)
Supplement: Attachment — Submitted filename: Response to reviewers_2nd Revision_HK.docx [file pgen.1009715.s016.docx]

Dear Dr. Gregory P. Copenhaver and Gregory Barsh,

Thank you very much for allowing us the opportunity to submit the revision of ‘Histone demethylase AMX-1 is necessary for proper sensitivity to interstrand crosslink DNA damage’ for publication in *PLoS* *Genetics*. **We incorporated all of the suggestions made by Reviewer 1.** The revised text further strengthens our original findings. We hope that the corrections will meet with your approval for this journal. All page numbers refer to the revised manuscript file with tracked changes. The responses to the reviewer’s comments are highlighted in blue below:

Reviewer #1:

1. While the authors now cite the report by Wang et al., 2020 (Nat Struct Mol Biol) there are still several places in the manuscript where they describe axm-1 as “previously uncharacterized” and “has not been investigated”. This is particularly evident in the Abstract, Author Summary, Introduction and Results. I ask that the authors carefully go through the manuscript and change these statements as appropriate.

Thank you for pointing this out. We have revised the manuscript and now it reads as follows:

Pg 2) AMX-1 is a histone demethylase and mainly localizes to embryonic cells, the mitotic gut and sheath cells.

Pg 3) AMX-1 mainly localizes to embryonic cells, the mitotic gut and sheath cells.

Pg 5) However, the functions of the mammalian LSD2 homolog, AMX-1, are not as well understood.

Pg 5) Here we have identified a role for the histone demethylase AMX-1, a previously uncharacterized protein conserved from worms through humans, in the DNA interstrand crosslink (ICL) repair in *C. elegans* for the first time.

Pg 6) AMX-1 is a conserved histone demethylase

Pg 21) In summary, our study revealed that AMX-1 is required for proper fertility, embryonic survival and adequate regulation of H3K4me2 in both mitotic and meiotic nuclei in *C. elegans*.

Pg 21) ~~However, the role for the histone demethylase homolog AMX-1 in DNA damage repair had never been investigated.~~  (The text has been removed to meet the reviewer’s request.)

2. In several places, the authors state that AMX-1 localizes to the embryo, gut and sheath cells. While I understand what they mean, “embryo” is not parallel to “gut and sheath cells”, please reword.

Thank you for pointing this out. We have revised the manuscript and now it reads as follows:

Pg 2) AMX-1 is a histone demethylase and mainly localizes to embryonic cells, the mitotic gut and sheath cells.

Pg 3) AMX-1 mainly localizes to embryonic cells, the mitotic gut and sheath cells.

Pg 5) We show that AMX-1 mainly localizes to embryonic, the mitotic gut and sheath cells, suggesting a role in mitotic nuclei.

Pg 8) Consistent with the phenotypic results, AMX-1-GFP signal is mainly observed in the nuclei of gut cells, embryonic cells (regardless of their stage), and sheath cells.

Pg 17) Consistently, immunostaining results found that AMX-1 expression increased upon lack of SPR-5 in the mitotic embryonic cells and gut cells, where the two histone demethylases overlap (Fig 3A).

Pg 17) lack of AMX-1 expression resulted in accumulation of H3K4me2 in embryonic cells and germline nuclei (Fig 4).

Pg 17) Similarly, no distinct changes were observed in embryonic cells or pachytene nuclei between single and double mutants.

Pg 18) Specifically, the expression of AMX-1 increases upon lack of SPR-5 in gut and embryonic cells (Fig 3).

Pg 32) Fig 3. AMX-1 is expressed in the nuclei of gut, embryonic and sheath cells and is further upregulated in absence of SPR-5. **(A)** Immunolocalization of AMX-1-GFP utilizing an anti-GFP antibody in the indicated mitotic cells and at different stages in the gonads of control (N2 worms), wild type (*amx-1::GFP* worms) and *spr-5; amx-1::GFP* worms. AMX-1-GFP signal is detected in the nuclei of mitotic cells including gut, embryonic and sheath cells.

Pg 33) **A)** The DNA damage checkpoint is activated in *amx-1* mutants, as shown by the increased pCHK-1 signal observed in embryonic cells, premeiotic tip-transition zone (TZ) and pachytene nuclei.

Pg 35) Bottom, in the absence of AMX-1, SPR-5 expression is up-regulated.

**3** While the authors clearly show that inactivation of either amx-1 or spr-5 lead to elevated expression levels of the other demethylase, at several places in the manuscript they say that they compensate for each other. Expression does not equate to function and unfortunately, the authors did not examine the phenotype of the amx-1; spr-5 double mutant (except looking at H3K4me levels). In the absence of that data, I do not think the authors should say that they compensate for each other, especially since they do have several different phenotypes as documented in this paper.

Thank you very much and we have revised the manuscript and now it reads as follows:

Pg 2) Expression of AMX-1 and of the histone H3K4 demethylase SPR-5 is reciprocally up-regulated upon lack of each other

Pg 5) Although AMX-1 and SPR-5 exhibit reciprocal up-regulated expression upon lack of each other, they are not fully functionally redundant.

Pg 8) Lack of either AMX-1 or SPR-5 results in reciprocal up-regulated expression in mitotic cells.

Pg 9) Consistent with our immunostaining results, this mRNA expression pattern suggests that AMX-1 expressed in mitotic cells might compensate, at least in part, for the lack of SPR-5.

Pg 9) Given that AMX-1 contains a conserved amine oxidase domain and is up-regulated upon lack of SPR-5 expression, we next examined AMX-1 histone demethylase activity *in vivo* by staining for dimethylated H3K4 level.

Pg 17) AMX-1 and SPR-5 exhibit reciprocal up-regulated expression in mitotic cells, but are not fully functionally redundant

Reviewer #2: The authors responded my all comments properly. I think this manuscript will be acceptable for publication.

Reviewer #3: Thank you for doing a terrific job of addressing all reviewer comments; it was a pleasure to read this manuscript!
